# 在线二维高效液相色谱-四极杆飞行时间质谱法分析两性霉素B的杂质谱

**DOI:** 10.3724/SP.J.1123.2023.07012

**Published:** 2024-05-08

**Authors:** Rongwen WENG, Xuantang WANG, Hongliang WEN, Hao LIU

**Affiliations:** 1.复旦大学药学院, 上海 201203; 1. College of Pharmacy, Fudan University, Shanghai 201203, China; 2.上海市食品药品检验研究院, 上海 201203; 2. Shanghai Institute for Food and Drug Control, Shanghai 201203, China; 3.中国医药工业研究总院, 上海 201203; 3. China State Institute of Pharmaceutical Industry, Shanghai 201203, China

**Keywords:** 在线二维高效液相色谱-四极杆飞行时间质谱, 两性霉素B, 杂质谱, on-line two-dimensional high performance liquid chromatography-quadrupole time-of-flight mass spectrometry (2D HPLC-Q TOF/MS), amphotericin B, impurity profile

## Abstract

建立了基于在线二维高效液相色谱-四极杆飞行时间质谱(2D HPLC-Q TOF/MS)的两性霉素B杂质谱分析方法。第一维液相色谱(^1^D-LC)以XBridge Shield C18为色谱柱、甲醇-乙腈-4.2 g/L柠檬酸水溶液为流动相。第二维液相色谱(^2^D-LC)以Xtimate C8为捕集柱,以含0.1%甲酸的10 mmol/L甲酸铵水溶液-乙腈(95∶5, v/v)为流动相进行捕集和脱盐;以Xtimate C8为色谱柱,以含0.1%甲酸的10 mmol/L甲酸铵水溶液-乙腈为流动相进行洗脱。该方法结合了在线稀释和多针捕集装置,实现了10个组分/杂质的高效分离,并通过四极杆飞行时间质谱实现了对不稳定组分/杂质的结构推断,解决了2D HPLC溶剂兼容性的难题,增加了分析通量,提高了2D HPLC的自动化能力,避免了两性霉素B与部分杂质在色谱分离过程中相互转化的问题,提高了检测灵敏度。利用该方法可以灵敏地对两性霉素B的杂质谱进行分析,所得到的研究结果对两性霉素B的生产工艺改进具有指导意义。

## Abstract (en)

Amphotericin B (AmB) is a polyene-macrolide antimicrobial drug with a broad antibacterial spectrum and remarkable efficacy against deep fungal infections. It binds to ergosterol on the fungal cell membrane and alters its permeability, thereby destroying the membrane. AmB is a multicomponent antimicrobial medication that contains a wide range of impurities, rendering quality analysis extremely difficult. In the current Chinese Pharmacopoeia (Edition 2020) and European Pharmacopoeia (EP10.3), high performance liquid chromatography (HPLC) is applied to examine related substances in AmB. However, this technique presents a number of issues. For instance, the mobile phases used in the HPLC method described in both references contain nonvolatile inorganic salts, which cannot be coupled with a mass spectrometry (MS) detector. In addition, because the mobile phases used have a low pH, the component/impurities of AmB drug can easily be degraded or interconverted during the analytical process, leading to reduced analytical accuracy. Therefore, the accuracy and sensitivity of this method must be improved. In this study, a method based on on-line two-dimensional high performance liquid chromatography-quadrupole time-of-flight mass spectrometry (2D HPLC-Q TOF/MS) was developed to analyze the impurity profile of AmB in accordance with the Chinese Pharmacopoeia (Edition 2020) and European Pharmacopoeia (EP10.3). The method combines on-line dilution and a multiple-capture HPLC system to achieve the efficient separation of AmB component/impurities. It also resolves the issue of poor solvent compatibility in 2D HPLC, increases the analytical flux, enhances the automation capability, reduces the mutual conversion of AmB and its impurities during the analytical process, and increases the detection sensitivity of the method. MS was also used to determine the structural inference of unstable components and impurities. An XBridge Shield C18 column (250 mm×4.6 mm, 3 μm) was used for first-dimensional-liquid chromatography with gradient elution using methanol-acetonitrile-4.2 g/L citric acid monohydrate solution (10∶30∶60, v/v/v, pH 4.7) as mobile phase A and methanol-acetonitrile-4.2 g/L citric acid monohydrate solution (12∶68∶20, v/v/v, pH 3.9) as mobile phase B. An Xtimate C8 column (10 mm×2.1 mm, 5 μm) was used as the trap column, and trapping and desalting were performed using 10 mmol/L ammonium formate aqueous solution containing 0.1% formic acid-acetonitrile (95∶5, v/v). An Xtimate C8 column (250 mm×2.1 mm, 5 μm) was used for second-dimensional-liquid chromatography with gradient elution using 10 mmol/L ammonium formate aqueous solution containing 0.1% formic acid-acetonitrile (95∶5, v/v) and 10 mmol/L ammonium formate aqueous solution containing 0.1% formic acid-acetonitrile (5∶95, v/v) as mobile phases. The data were collected in positive-ion mode. In this study, the structures of six impurities in amphotericin B were inferred, according to the fragmentation, the MS and MS^2^ spectra of each impurity. The developed method can be used to quickly and sensitively analyze the impurity profile of AmB. Furthermore, the research results on impurity profiles can be applied to guide improvements in AmB production.

两性霉素B(amphotericin B, AmB)是一种多烯大环内酯类微生物药物,具有广谱抗真菌作用^[1]^。近年来,两性霉素B的临床应用越来越广泛^[2]^,但两性霉素B中存在多种组分和杂质,这些物质可能与其副作用相关^[3]^。目前,研究者们致力于探索能够抑制两性霉素B副作用的新途径,例如将两性霉素B与其他抗生素联合使用、设计新的剂型、提高给药效率等^[4]^。

传统的高效液相色谱已广泛应用于两性霉素B样品组分/杂质的分离分析中^[5]^,但分离效果并不理想。中国药典(2020版)^[5]^和欧洲药典(EP10.3)^[6]^采用反相液相色谱法(RPLC)对两性霉素B样品中的有关物质进行控制,但二者采用的流动相均含有非挥发性无机盐,无法与质谱检测器进行联用;并且二者所采用的流动相pH值较低,两性霉素B样品的组分/杂质容易在分析过程中发生降解和相互转化,导致分析准确性降低^[7]^。二维高效液相色谱(two-dimensional high performance liquid chromatography, 2D HPLC)通过组合具有不同分离机制的色谱柱,展现出了峰容量大、分辨率高和分离能力强等优势^[8,9]^。通过正相液相色谱(NPLC)与RPLC的组合^[10,11]^、亲水作用色谱(HILIC)与RPLC的组合^[12]^、体积排阻色谱(SEC)与RPLC的组合^[13,14]^、离子交换色谱(IEC)与RPLC的组合^[15,16]^、超临界流体色谱(SFC)与RPLC的组合^[17]^等可以达到更高的分离选择性和检测灵敏度,从而实现复杂微生物药物中多个组分/杂质的高效分离和分析^[18,19]^。质谱技术可用于化合物的结构鉴定,将二维液相色谱与质谱技术联用能够同时发挥二者的优势,且二维液相色谱-质谱已在药物分析领域得到了广泛的应用^[17,20,21]^。

基于以上情况,本研究通过在线二维高效液相色谱-四极杆飞行时间质谱(2D HPLC-Q TOF/MS)实现了两性霉素B样品中10个组分/杂质的高效分离,并提高了结构鉴定的准确性,可用于全面了解两性霉素B样品中组分/杂质的结构特征,为两性霉素B的质量控制提供了科学依据。

## 1 实验部分

### 1.1 仪器、试剂与材料

第一维液相色谱(^1^D-LC)系统:Agilent 1260 Infinity液相色谱分离模块,Agilent 1290 Infinity光电二极管阵列检测器;第二维液相色谱(^2^D-LC)系统:Agilent 1290 Infinity高效液相色谱仪,Agilent 1260 Infinity二极管阵列检测器; Agilent 6550 iFunnel Q-TOF质谱仪,以上仪器均购自美国Agilent公司。

两性霉素B原料药(效价1024单位/mg)、1*R*-氧-甲基-两性霉素B(纯度99.6%)、1-氧-乙基-两性霉素B(纯度99.5%)和1*S*-氧-甲基-两性霉素B(纯度99.6%)均购于河北亚诺生物科技股份有限公司;乙腈(质谱级)、甲醇(质谱级)、甲酸(质谱级)、甲酸铵(质谱级)、柠檬酸(优级纯)、乙酸铵(质谱级)、*N*-甲基吡咯烷酮(质谱级)(德国Merck公司);实验所用超纯水由Milli-Q净水系统纯化后获得。

### 1.2 供试品溶液的配制

将20.0 mg两性霉素B原料药溶解于15.0 mL *N*-甲基吡咯烷酮中,再用10 g/L乙酸铵水溶液-*N*-甲基吡咯烷酮-甲醇(1∶1∶2, v/v/v)稀释至50.0 mL,摇匀;取5.0 mL上述溶液,用10 g/L乙酸铵水溶液-*N*-甲基吡咯烷酮-甲醇(1∶1∶2, v/v/v)稀释至25.0 mL,配制成质量浓度为0.08 mg/L的供试品溶液。

### 1.3 分析条件

#### 1.3.1 ^1^D-LC条件

色谱柱:Waters XBridge Shield RP18柱(250 mm×4.6 mm, 3 μm),C18填料孔径为13 nm;柱温:25 ℃;流速:0.8 mL/min;进样量:20 μL;检测波长:383 nm。流动相A为甲醇-乙腈-4.2 g/L柠檬酸水溶液(10∶30∶60, v/v/v, pH 4.7),流动相B为甲醇-乙腈-4.2 g/L檬酸酸水溶液(12∶68∶20, v/v/v, pH 3.9),其中流动相的pH值用浓氨水调节。线性梯度洗脱程序:0~4 min, 0B~4%B; 4~22 min, 4%B~7%B; 22~32 min, 7%B~77%B; 32~32.1 min, 77%B~95%B; 32.1~38 min, 95%B; 38~38.1 min, 95%B~0B; 38.1~46 min, 0B。

#### 1.3.2 ^2^D-LC条件

捕集柱:Welch Xtimate C8柱(10 mm×2.1 mm, 5 μm);流速:0.5 mL/min;稀释4倍,累计进样4次;流动相为含0.1%甲酸的10 mmol/L甲酸铵水溶液-乙腈(95∶5, v/v)。

色谱柱:Welch Xtimate C8柱(250 mm×2.1 mm, 5 μm),C8填料孔径为30 nm;柱温:25 ℃;流速:0.2 mL/min;检测波长:383 nm;流动相A为含0.1%甲酸的10 mmol/L甲酸铵水溶液-乙腈(95∶5, v/v),流动相B为含0.1%甲酸的10 mmol/L甲酸铵水溶液-乙腈(5∶95, v/v)。线性梯度洗脱程序:0~1 min, 5%B; 1~5 min, 5%B~17%B; 5~35 min, 17%B~27%B; 35~65 min, 27%B~30%B; 65~80 min, 30%B~45%B; 80~81 min, 45%B~95%B; 81~90 min, 95%B; 90~90.1 min, 95%B~5%B; 90.1~105 min, 5%B。根据切割馏分的保留时间,可以对^2^D-LC的梯度程序进行适当修改,以提高分离效率。

#### 1.3.3 质谱条件

离子源:电喷雾电离(ESI)源,喷针水平角度45°;采用不分流进样,鞘气流速:10 L/min;进样时间:10~60 min;离子源温度:350 ℃;喷雾电压:3.5 kV;加热毛细管温度:320 ℃;采集模式:正离子模式;碎裂能量:35 eV; MS^1^数据采集范围:*m/z* 100~2000;使用数据依赖型采集模式,取MS^1^强度排名前3的离子进行MS^2^碎裂采集;MS^2^数据采集范围:*m/z* 300~1500。

### 1.4 分析过程

首先根据^1^D-LC的紫外检测图谱来确定目标杂质的保留时间,再分别对各杂质进行多次中心切割,通过阀切换技术,对目标杂质进行在线稀释,并将其富集至捕集柱上,同时进行脱盐操作;之后将杂质从捕集柱转移至^2^D-LC进行分离和质谱分析;根据两性霉素B的基本结构以及MS^1^和MS^2^谱图,再结合碎裂规律等信息来推导各杂质的可能结构。

## 2 结果与讨论

### 2.1 2D HPLC分离装置的搭建及运行

设计并搭建了2D HPLC-Q TOF/MS装置,装置图如图1所示。两性霉素B样品的2D HPLC分离以在线方式进行,即先使用^1^D-LC对两性霉素B样品进行分离,再通过阀切换将目标馏分切入至定量环中,同时运行补液泵,以对目标馏分进行在线稀释,使其能被捕集柱高效捕集;通过^2^D-LC对目标馏分进行再次分离,并使用质谱进行在线检测。

**图1:**
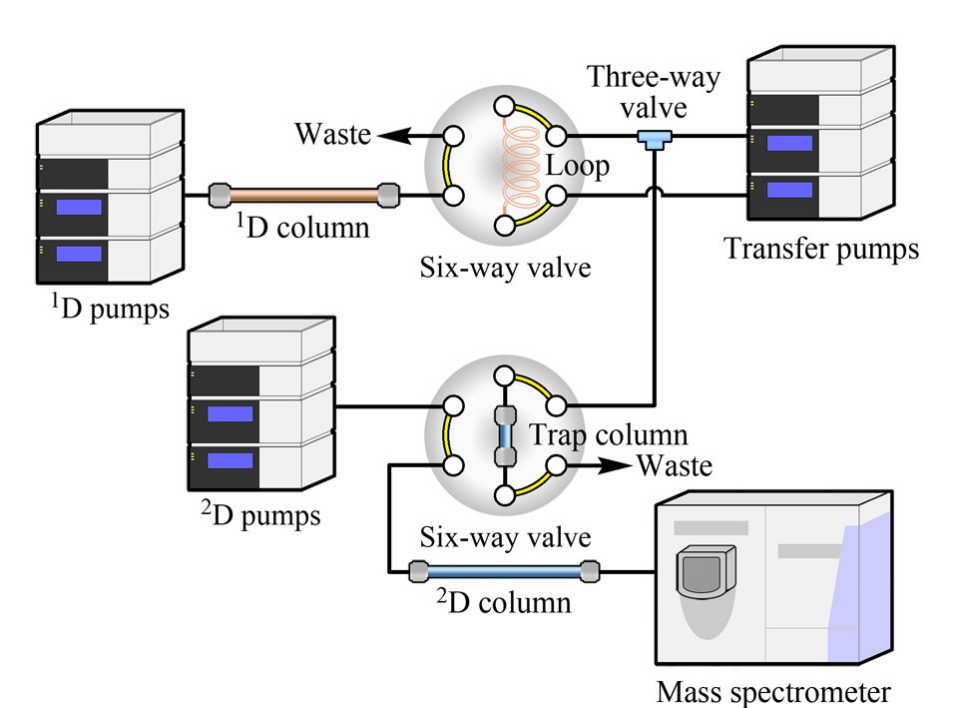
在线2D-HPLC-Q TOF/MS装置图

在^1^D-LC中,由于流动相中有机相的比例较高,可能导致馏分无法被捕集柱充分捕集,故加入了在线稀释装置。在线稀释装置主要由补液泵和一个三通阀组成,补液泵通过将^2^D-LC的流动相A以0.5 mL/min流速泵出,再通过三通阀与^1^D-LC的流出液进行混合稀释,降低了有机相比例,最终目标馏分内含有体积分数为6%~10%的乙腈,随后被捕集柱捕集,进行^2^D-LC分离。

### 2.2 ^1^D-LC分析

两性霉素B供试品溶液在^1^D-LC条件下的紫外检测图谱如图2所示,将其中归一化峰面积大于0.15%或含有已知组分/杂质的色谱峰及其峰面积占比进行归纳,结果见表1;其中,色谱峰1~7及9~10对应为杂质,色谱峰8对应为两性霉素B。

**图2:**
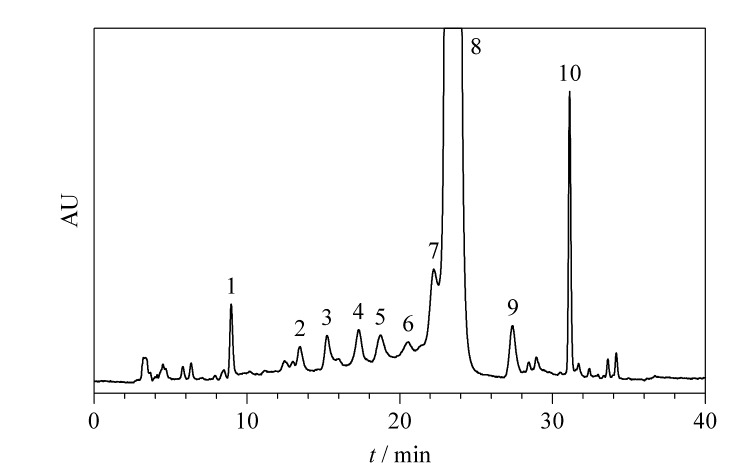
两性霉素B供试品溶液在^1^D-LC条件下的色谱图

**表1:** 两性霉素B供试品溶液在^1^D-LC条件下的保留时间及归一化峰面积

No.	t_R_/min	Normalized peak area/%
1	8.97	0.38
2	13.48	0.16
3	15.24	0.26
4	17.30	0.33
5	18.74	0.32
6	20.57	0.14
7	23.22	1.70
8	23.44	94.96
9	27.39	0.55
10	31.12	1.20

The Nos. are the same as in Fig. 1.

### 2.3 ^2^D-LC条件优化

#### 2.3.1 色谱柱及流动相的选择

^1^D-LC中所使用的流动相含有非挥发性盐,导致^1^D-LC难以直接与质谱联用,因此需引入^2^D-LC,以对^1^D-LC的馏分进行除盐,从而实现与质谱的联用分析。为实现^2^D-LC的高效分离及质谱在线检测,在^2^D-LC中仍使用反相液相色谱模式以达到快速分离和高效离子化的目的。^1^D-LC使用孔径为13 nm的C18填料,^2^D-LC使用孔径为30 nm的C8填料,以及二者不同的流动相,以实现二维RPLC-RPLC的高正交性分离。药物分析中常用的色谱柱内径通常为4.6 mm,其对应的最佳流动相流速为0.8~1.0 mL/min,但该流速大于质谱ESI源所对应的最佳流速,因此需要进行分流,但分流操作必然会导致分析灵敏度降低。因此^2^D-LC选用内径为2.1 mm的色谱柱,其最佳流速为0.2 mL/min,可直接接入质谱离子源进行分析。^2^D-LC中色谱柱的进样量较小,引入与^2^D-LC色谱柱填料粒径相同但长度(10 mm)较短的捕集柱,将大体积样品溶液以较高流速注入捕集柱,以达到增大进样量、提高灵敏度的目的。

本研究在^1^D-LC中采用C18色谱柱,在该色谱柱条件下,两性霉素B在低比例有机相下已被洗脱,为了提高二维正交性,^2^D-LC选用疏水性较弱的C8色谱柱。两性霉素B在稳定构象下的分子直径约为2~3 nm,首先通过预实验来考察不同孔径(13 nm和30 nm)的C8色谱柱对两性霉素B组分/杂质分离效果的影响,结果表明,在^2^D-LC中使用大孔径的C8色谱柱有助于获得更好的分离正交性,因此^2^D-LC选用孔径为30 nm的C8色谱柱。

为实现^2^D-LC的高效分离及其流出液在质谱离子源中的高效离子化,在^2^D-LC流动相中加入甲酸;同时,为了避免两性霉素B样品中的组分/杂质在色谱分离过程中产生酸降解,在流动相中加入甲酸铵水溶液。

#### 2.3.2 在线稀释及捕集条件优化

为实现目标馏分的高效捕集,对不同流动相流速、稀释倍数及进样次数下两性霉素B组分/杂质的检测灵敏度进行了考察。在相同进样次数及稀释倍数下,以两性霉素B色谱峰的峰面积为考察指标,结果如图3a所示,随着流速的增加,两性霉素B的峰面积呈现先增加后降低的趋势,在流速达到0.5 mL/min时,峰面积最高。在相同进样次数及流速下,随着稀释倍数的增加,两性霉素B的峰面积也逐渐增加,当稀释倍数为4倍时,两性霉素B的峰面积最高(图3b)。增加进样次数会直接导致分析时间的延长,杂质2的化学性质较稳定,且保留弱,洗脱快,因此本实验将杂质2的色谱峰面积作为考察指标,以节省进样次数的优化时间。在相同流速及稀释倍数下,随着进样次数的增多,杂质2的检测灵敏度不断升高,结果见图3c。综上,考虑分析时间及检测灵敏度,确定实验的最佳在线稀释及捕集条件如下:累计进样4次,稀释4倍,以0.5 mL/min的流速反冲至捕集柱。

**图3:**
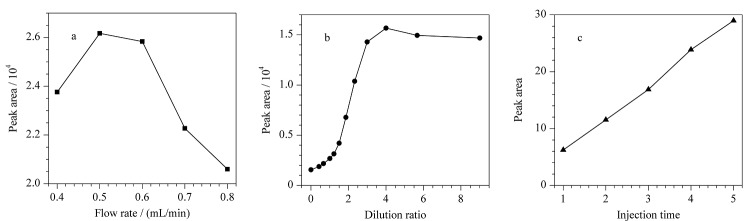
(a)流动相流速、(b)稀释倍数对两性霉素B色谱峰面积的影响及(c)进样次数对杂质2色谱峰面积的影响

采用中国药典(2020版)方法,^1^D-LC无法与质谱直接联用,而将^1^D-LC与^2^D-LC相接时,馏分在^2^D-LC分析过程中易发生降解,检测灵敏度降低,难以进行后续质谱分析。因此,本研究通过组合在线稀释及多针捕集装置来提高检测灵敏度,并通过与质谱联用来实现对未知杂质的结构解析。与中国药典(2020版)方法相比,本方法避免了杂质2~7在分析过程中的降解,具有较高的分析准确性。

### 2.4 组分/杂质结构的推断

分析比较10个组分/杂质经^2^D-LC分离后的色谱图,结果如图4所示,待测组分和杂质间的分离度较好,但各杂质在分析过程中均存在一定程度的降解,其中杂质1完全降解为主成分两性霉素B,杂质2和7也发生了一定程度的降解,杂质9和10的降解程度较小;通过对比^1^D-LC条件下的色谱图与欧洲药典(EP10.3)标准图谱,推断杂质1为已知杂质1*R*-氧-甲基-两性霉素B或1*S*-氧-甲基-两性霉素B,二者的结构图如图5所示,图5中的另一杂质1-氧-乙基-两性霉素B为中国药典(2020版)方法中包含的已知杂质,在本研究中仅作确认用;杂质3和6的质谱信息较少,无法对其结构进行推断,故本研究对杂质2、4、5、7、9、10共6个成分进行结构推断。

**图4:**
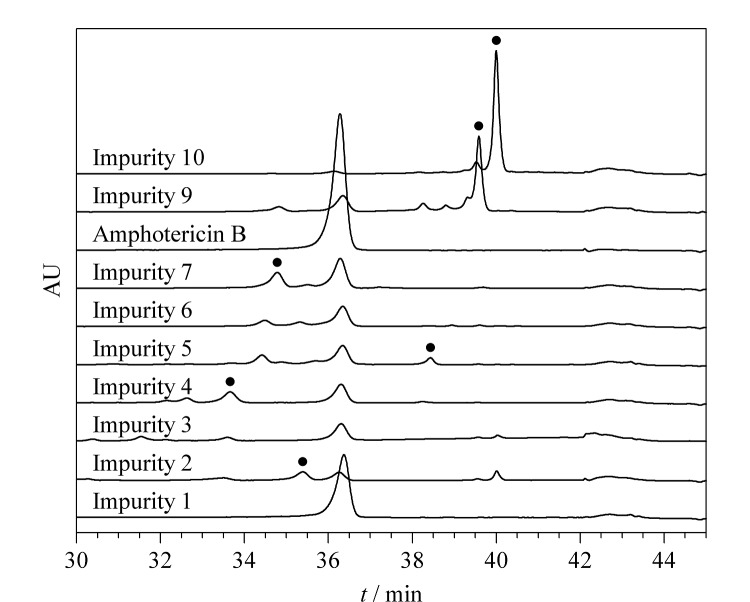
两性霉素B供试品溶液在^2^D-LC条件下的色谱图

**图5:**
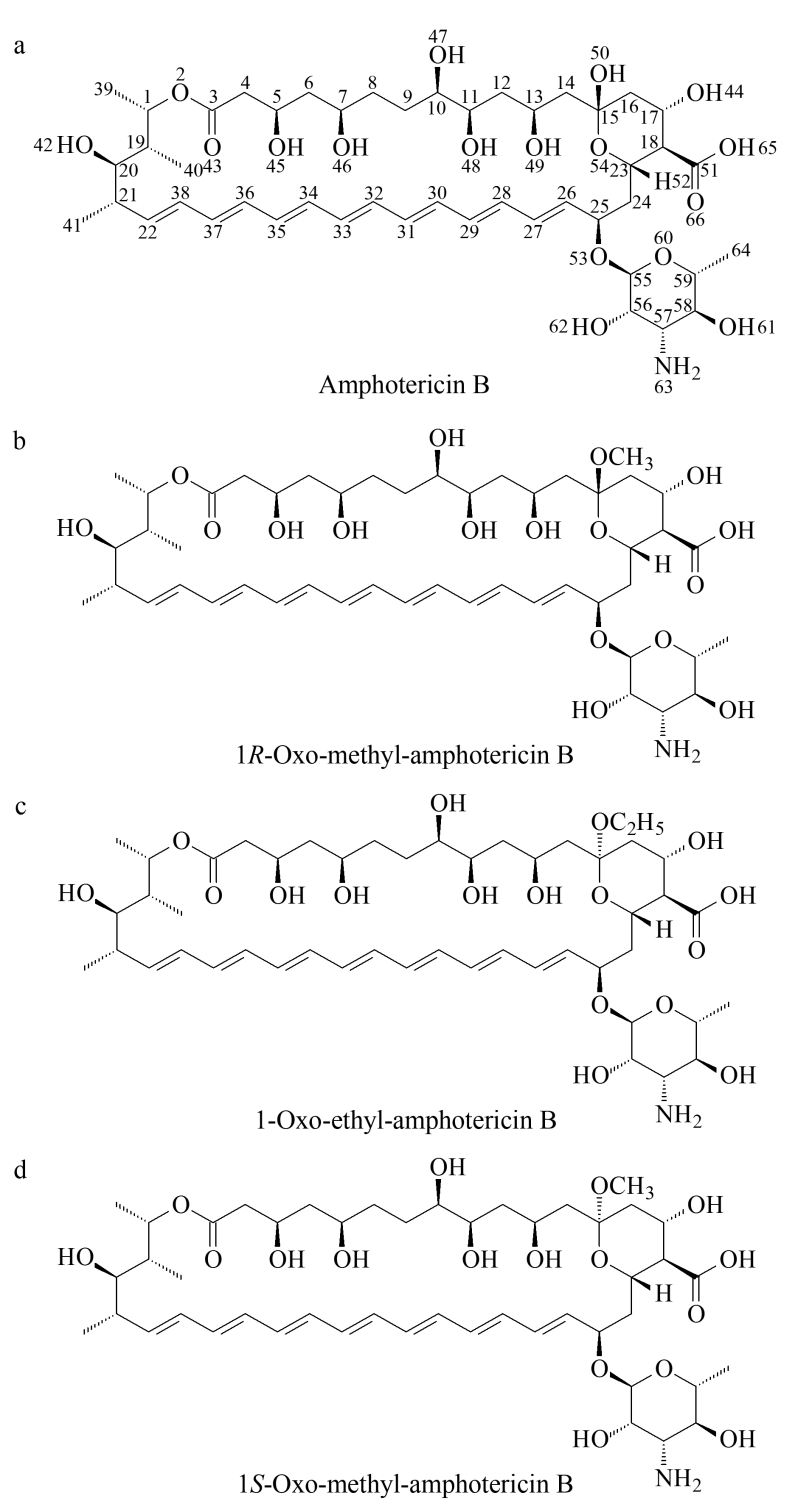
(a)两性霉素B、(b)杂质1*R*-氧-甲基-两性霉素B、(c)杂质1-氧-乙基-两性霉素B和(d)杂质1*S*-氧-甲基-两性霉素B的结构式

首先,对两性霉素B的碎裂规律进行推断,两性霉素B的一级质谱图如图6a所示,其准分子离子峰为[M+H]^+^(*m/z* 924.4341),推断为两性霉素B分子;*m/z* 946.4120处的质谱峰为两性霉素B分子与钠离子结合后产生的加钠峰([M+Na]^+^), *m/z* 906.4252处的质谱峰为两性霉素B分子脱去1个水分子后产生的,继续脱去氨糖基团后产生*m/z* 761.3564碎片。

**图6:**
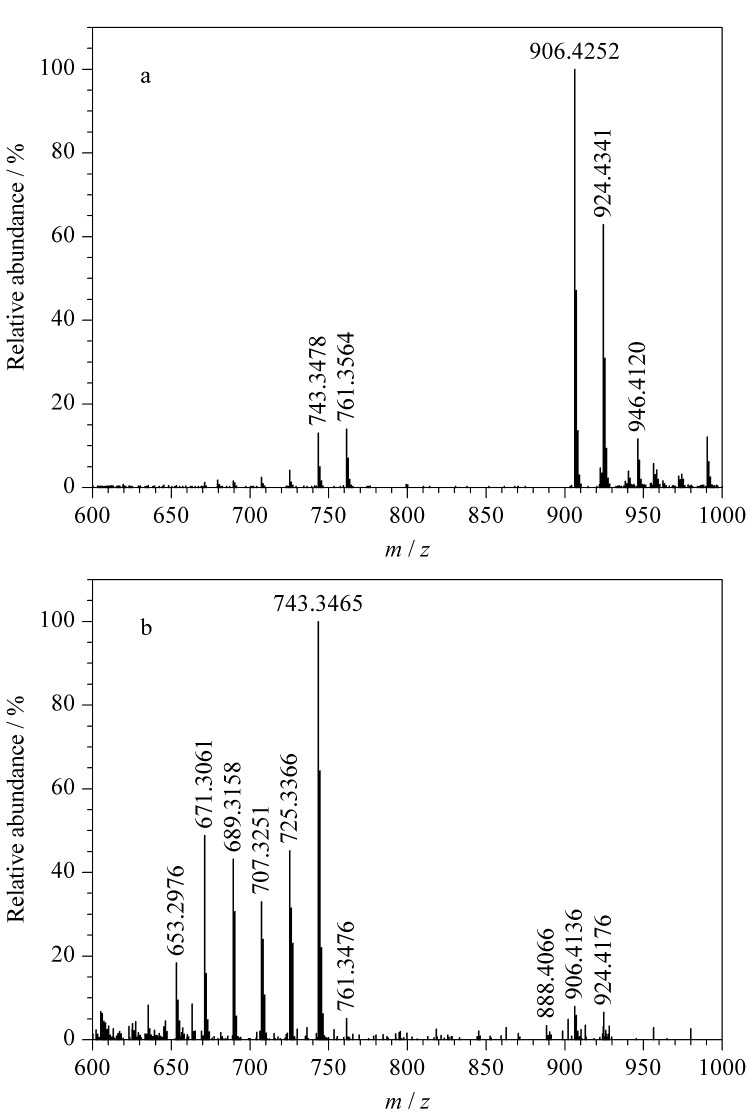
两性霉素B的(a)一级和(b)二级质谱图

由两性霉素B的二级质谱图(图6b)可知,两性霉素B分子在脱去1个水分子后生成*m/z* 906.4136碎片,该碎片再脱去1个水分子后得到*m/z* 888.4066碎片,进一步脱去氨糖基团生成*m/z* 743.3465碎片;*m/z* 906.4136碎片脱去氨糖基团后生成*m/z* 761.3476碎片,进一步脱去1个水分子得到*m/z* 743.3465碎片,该碎片多次脱去1个水分子后依次形成*m/z* 725.3366、 707.3251、 689.3158、 671.3061、 653.2976等碎片,两性霉素B的碎裂过程见图7。

**图7:**
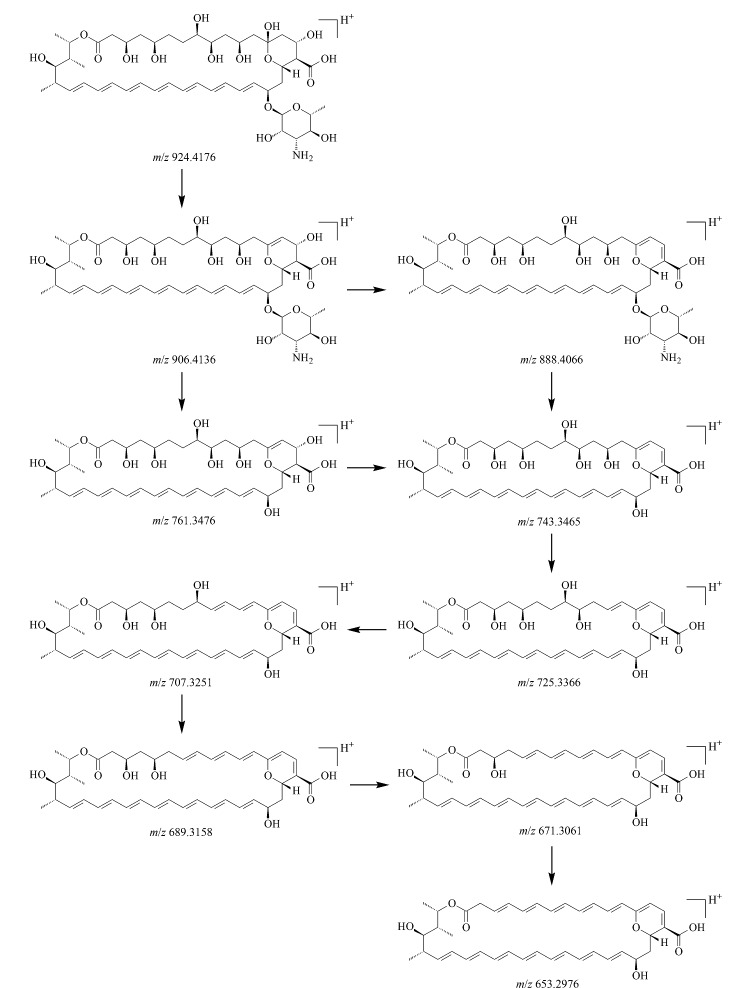
两性霉素B的碎裂规律

杂质2的质谱图与两性霉素B的质谱图无明显差异,其准分子离子峰为[M+H]^+^(*m/z* 924.4325),与钠离子结合后产生的加钠峰([M+Na]^+^)*m/z* 为946.4099,脱去1个水分子后产生*m/z* 906.4222碎片,脱去氨糖基团后产生*m/z* 761.3565碎片,故推测杂质2可能为两性霉素B分子的同分异构体,分子式为C_47_H_73_NO_17_。

由杂质4的一级质谱图(图8a)可知,其准分子离子峰为[M+H]^+^(*m/z* 922.4152), *m/z* 904.4116处的质谱峰为杂质4脱去1个水分子后产生的,*m/z* 759.3415处的质谱峰为杂质4脱去氨糖基团后产生的,在此基础上进一步脱去1个水分子后产生*m/z* 741.3338碎片;由此推断,杂质4为两性霉素B分子内增加了一个双键后的化合物,其中增加的双键可能是碳-碳双键,也可能是两性霉素B分子结构中的一个羟基转化为了羰基。为进一步确定杂质4的结构,对杂质4的二级质谱图(母离子为*m/z* 904.3574)进行分析。如图8b所示,杂质4脱去1个水分子后生成*m/z* 886.3938碎片,进一步脱去氨糖基团后生成*m/z* 741.3270碎片,该碎片多次脱去1个水分子后依次形成*m/z* 723.3175、705.3216、687.3121、669.2890、651.2709等碎片。值得注意的是,杂质4可以脱去完整的氨糖片段(*m/z* 145),由此判断杂质4中增加的双键位于内酯大环之上。两性霉素B分子中17位点处的羟基活性较高(图5a),易脱落,也容易被氧化,因此推断杂质4中增加的双键可能来源于羟基氧化生成的羰基,杂质4的分子式为C_47_H_72_NO_17_,碎裂过程见图9。杂质5的质谱图与杂质4相似,其准分子离子峰为[M+H]^+^(*m/z* 922.4183),脱去1个水分子后生成*m/z* 904.4088碎片,脱去氨糖基团后生成*m/z* 759.3403碎片,该碎片进一步脱去1个水分子后生成*m/z* 741.3284碎片,故推测杂质5与杂质4可能互为同分异构体,分子式同为C_47_H_72_NO_17_。

**图8:**
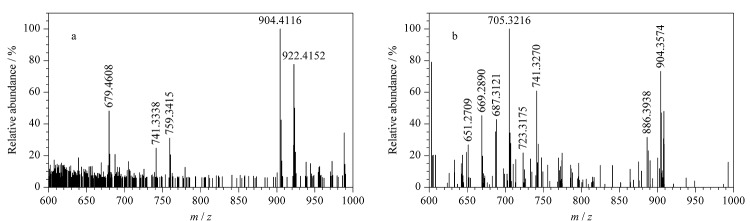
杂质4的(a)一级和(b)二级质谱图

**图9:**
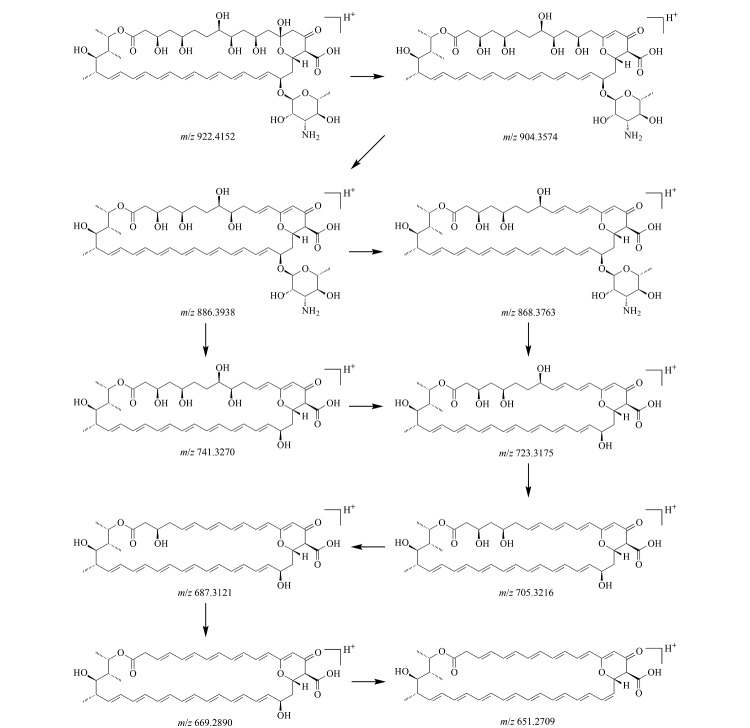
杂质4的碎裂规律

由杂质7的一级质谱图(图10a)可知,其准分子离子峰为[M+H]^+^(*m/z* 940.4244), *m/z* 962.4044处的质谱峰为其与钠离子结合后产生的加钠峰([M+Na]^+^), *m/z* 922.4180处的质谱峰为杂质7脱去1个水分子后所产生的,*m/z* 777.3488处的质谱峰为*m/z* 922.4180碎片脱去氨糖基团后产生的,进一步脱去1个水分子后生成*m/z* 759.3392碎片。为进一步确定杂质7的结构,对杂质7的二级质谱图(见图10b)进行分析,该母离子脱去1个水分子后生成*m/z* 904.8384碎片,该母离子再脱去一个氨糖基团生成*m/z* 777.3488碎片,进一步脱去1个水分子后得到*m/z* 759.3222碎片,之后多次脱去1个水分子后依次形成*m/z* 741.3275、723.3497、705.3097、687.3121、669.3305等碎片。由以上碎裂规律推断,杂质7是由两性霉素B分子先经水解再增加一个双键后产生的。通过考察杂质7的紫外吸收光谱,未见其主要吸收峰的波长位置发生变化,因此考虑其大环中的7个碳-碳双键共轭结构未发生变化,推测杂质7是由两性霉素B分子发生了酯键水解且17位点处的羟基被氧化为羰基后产生的。杂质7的分子式为C_47_H_73_NO_18_,碎裂过程见图11。

**图10:**
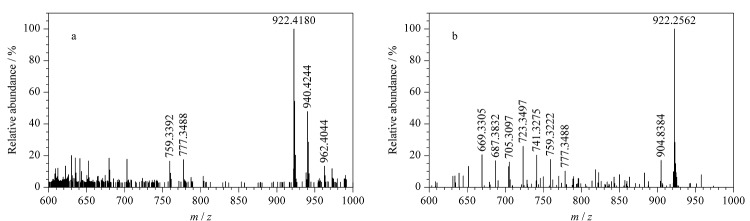
杂质7的(a)一级和(b)二级质谱图

**图11:**
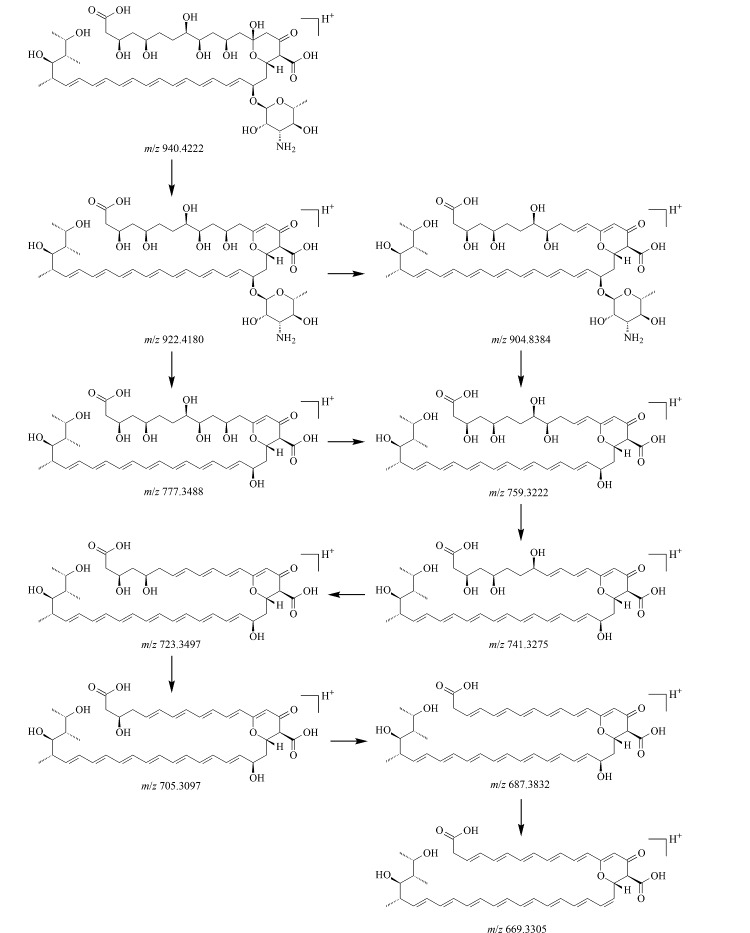
杂质7的碎裂规律

杂质9的准分子离子峰为[M+H]^+^(*m/z* 743.3498),推测其结构为两性霉素B分子脱去氨糖基团及2个水分子后的内酯大环结构。为进一步验证杂质9的结构,对其二级质谱图(母离子为*m/z* 743.4211,图12)进行分析,结果表明,杂质9的二级质谱图结果与上述推测的碎裂方式相符,因此推断杂质9为两性霉素B分子脱去氨糖基团及2个水分子后的产物,分子式为C_41_H_59_O_12_,碎裂过程见图13。

**图12:**
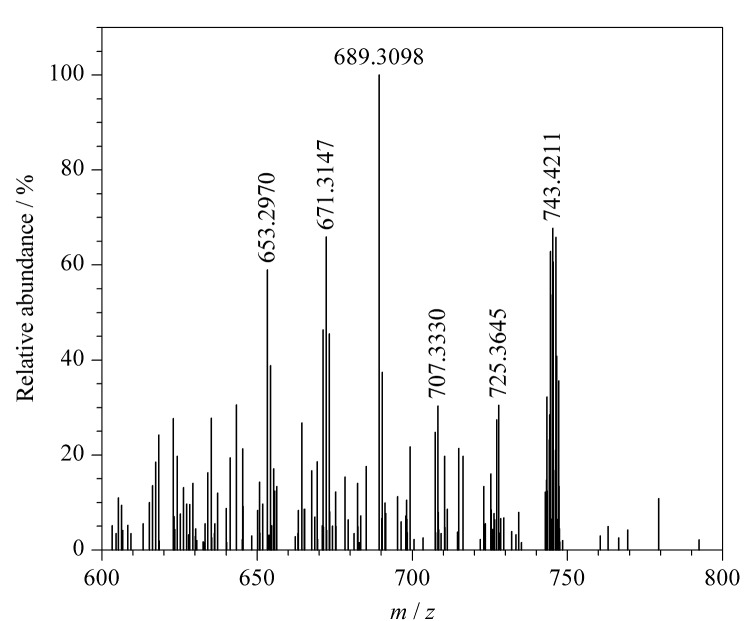
杂质9的二级质谱图

**图13:**
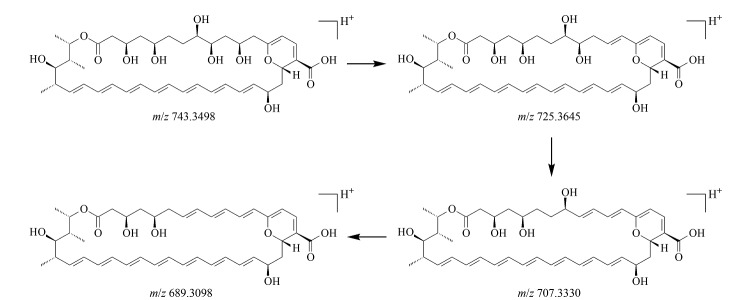
杂质9的碎裂规律

由杂质10的一级质谱图(图14a)可知,其准分子离子峰为[M+H]^+^(*m/z* 908.4392), *m/z* 930.4199处的质谱峰为其与钠离子结合后产生的加钠峰([M+Na]^+^), *m/z* 890.4307处的质谱峰为杂质10脱去1个水分子后产生的,*m/z* 745.3627处的质谱峰为杂质10脱去氨糖基团后产生的,进一步脱去1个水分子产生*m/z* 727.3531碎片。

**图14:**
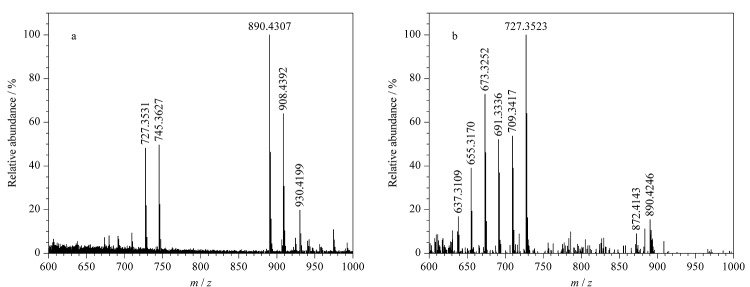
杂质10的(a)一级和(b)二级质谱图

杂质10的*m/z*与两性霉素B分子的*m/z*相差16,存在去羟基化的可能性。对杂质10的二级质谱图(见图14b)进行分析,杂质10的母离子脱去1个水分子生成*m/z* 872.4143碎片,继续脱去氨糖基团生成*m/z* 727.3523碎片;杂质10的母离子直接脱去氨糖基团生成*m/z* 745.3627碎片,再脱去1个水分子得到*m/z* 727.3523碎片,该碎片多次脱去1个水分子后依次形成*m/z* 709.3417、691.3336、673.3252、655.3170、637.3109等碎片。无论在母离子或子离子中,均存在脱去氨糖基团的现象,而在脱去氨糖基团后,杂质10的*m/z*与两性霉素B分子的*m/z*仍然相差16,因此推断杂质10的去羟基化位点存在于两性霉素B母环中而非氨糖基团中,结合文献[22]初步判定其分子式为C_47_H_74_NO_16_,碎裂规律如图15所示。

**图15:**
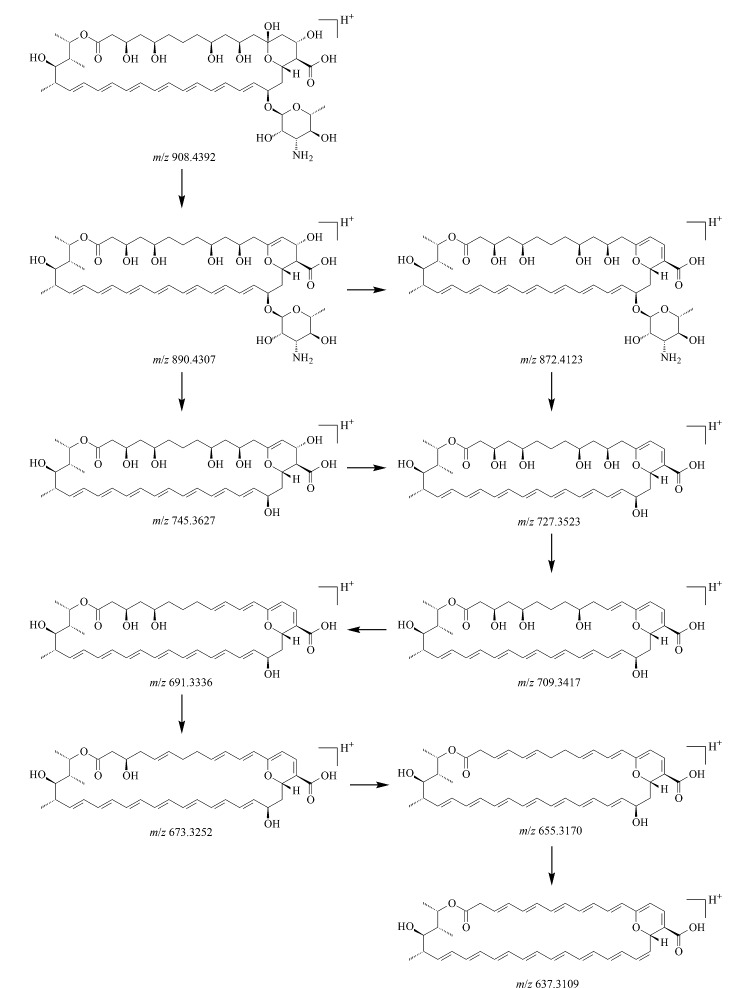
杂质10的碎裂规律

## 3 结论

本研究基于在线高效二维液相色谱-四极杆飞行时间质谱建立了两性霉素B杂质谱的分析方法。该方法解决了二维液相色谱的溶剂兼容性问题,增加了分析通量,提高了二维液相色谱的自动化能力,避免了两性霉素B与部分杂质在色谱分离过程中的相互转化,提高了检测灵敏度;通过与质谱技术联用,本研究实现了对不稳定杂质的结构推断。本研究共推断出了两性霉素B样品中6个杂质的可能结构,为后续两性霉素B的质量评价及杂质谱分析奠定了基础,同时也为其他微生物药物杂质谱研究提供了新的思路。由于本文中杂质结构鉴定的信息量较少,后续考虑对杂质进行制备,并采用核磁共振等技术进一步确证杂质的结构。
